# Methanol outbreak: a Malaysian tertiary hospital experience

**DOI:** 10.1186/s12245-020-0264-5

**Published:** 2020-02-07

**Authors:** J. Md Noor, R. Hawari, M. F. Mokhtar, S. J. Yussof, N. Chew, N. A. Norzan, R. Rahimi, Z. Ismail, S. Singh, J. Baladas, N. H. Hashim, M. I. K. Mohamad, M. D. Pathmanathan

**Affiliations:** 1 Emergency Department, Universiti Teknologi MARA, Jalan Hospital, 47000 Sungai Buloh, Selangor Malaysia; 2 Emergency & Trauma Department, Hospital Sg Buloh, Jalan Hospital, 47000 Sungai Buloh, Selangor Malaysia; 3 Department of Pathology, Universiti Teknologi MARA, Jalan Hospital, 47000 Sungai Buloh, Selangor Malaysia; 4 Department of Public Health & Preventative Medicine, Universiti Teknologi MARA, Jalan Hospital, 47000 Sungai Buloh, Selangor Malaysia; 5 National Institute of Health, Shah Alam, Selangor Malaysia

**Keywords:** Methanol, Toxicity, Poisoning, Outbreak, Dialysis

## Abstract

**Introduction:**

Methanol poisoning usually occurs in a cluster and initial diagnosis can be challenging. Mortality is high without immediate interventions. This paper describes a methanol poisoning outbreak and difficulties in managing a large number of patients with limited resources.

**Methodology:**

A retrospective analysis of a methanol poisoning outbreak in September 2018 was performed, describing patients who presented to a major tertiary referral centre.

**Result:**

A total of 31 patients were received over the period of 9 days. Thirty of them were males with a mean age of 32 years old. They were mostly foreigners. From the 31 patients, 19.3% were dead on arrival, 3.2% died in the emergency department and 38.7% survived and discharged. The overall mortality rate was 61.3%. Out of the 12 patients who survived, two patients had toxic optic neuropathy, and one patient had uveitis. The rest of the survivors did not have any long-term complications.

Osmolar gap and lactate had strong correlations with patient’s mortality. Serum pH, bicarbonate, lactate, potassium, anion gap, osmolar gap and measured serum osmolarity between the alive and dead patients were significant. Post-mortem findings of the brain were unremarkable.

**Conclusion:**

The mortality rate was higher, and the morbidity includes permanent visual impairment and severe neurological sequelae. Language barrier, severity of illness, late presentation, unavailability of intravenous ethanol and fomipezole and delayed dialysis may have been the contributing factors. Patient was managed based on clinical presentation. Laboratory parameters showed difference in median between group that survived and succumbed for pH, serum bicarbonate, lactate, potassium and osmolar and anion gap. Management of methanol toxicity outbreak in resource-limited area will benefit from a well-designed guideline that is adaptable to the locality.

## Introduction

Methanol, or commonly known as methyl alcohol, is a colourless fluid compound. It consists of carbon monoxide and hydrogen ions (CH_3_OH). Methanol is also referred to as methyl alcohol, wood alcohol or wood spirit. It is a volatile, colourless, flammable poisonous fluid, produced from distillation of destructed wood particles [1]. This compound has been illegally used in the production of cheap and counterfeit ethanol. In Malaysia, there exists a black market for counterfeit alcohol which was sold to foreign workers for a cheaper price.

Upon ingestion, formic acid, a by-product of methanol oxidation via alcohol dehydrogenase, causes multiple toxic manifestations including optic neuropathy, cerebral oedema, acute renal failure and severe metabolic acidosis. Symptoms may present as early as few hours or up to 2 days post ingestion [2]. Lethal dose has been reported as 1.2 mL/kg [3]. Methanol ingestion-related mortality ranges from 18 to 44%. Morbidity outcomes amongst survivors include permanent visual impairment and severe neurological sequelae [4, 5].

Methanol toxicity may present a challenging diagnosis. Patients may be obtunded and unable to offer history. Suspicions should arise in patients with severe unexplained metabolic acidosis [6, 7]. As measured blood methanol is not widely available, anion gap and serum osmolal gap have been advocated as tools to clinch the diagnosis. Fomepizole, a competitive inhibitor of alcohol dehydrogenase prevents the formation of alcohol metabolites, is the antidote of choice [2]. However, fomepizole is often very costly, making ethanol a common alternative treatment of choice [8]. Sodium bicarbonate and haemodialysis help to correct the acid-base disturbance. Finally, folinic acid potentially enhances the metabolism of formic acid [9].

Outcomes of methanol toxicity are closely related to the interval time between ingestion and initiation of therapy and severity of acidosis as opposed to the initial serum methanol levels [10].

Massive outbreaks are uncommon. During this outbreak, we were able to not only streamline the management, but we were also able to gather data regarding under a controlled condition. The objectives of this paper are to discuss the clinical presentations and epidemiology, case management and the pearls and pitfalls experienced by a tertiary medical centre. The data regarding the outcomes will be presented.

## Materials and methods

### Patients’ presentation

This is a retrospective analysis of a methanol poisoning outbreak in the state of Selangor, in September 2018. This case description refers to patients attended at Hospital Sungai Buloh, a 620-bedded major tertiary referral centre in the suburban area of Klang Valley, Malaysia. Between 15 and 21 September 2018, 31 patients arrived to our emergency department. Six were brought in dead, and one died in the emergency department within 3 h. Seven were transferred to six different facilities. These patients were transferred out due to constraints in intensive care unit (ICU) bed in our hospital. Those that were transferred out were deemed more stable than those that we admitted to our ICU. Out of 24 that received treatment, only 12 survived.

The initial encounters of methanol cases were undiagnosed by the attending physicians. The clinical manifestations did not arouse suspicion and seemed to greatly mimic other diagnoses. The first two patients presented with altered mental status and vomiting with associated blurring of vision were diagnosed as septic meningitis and high anion gap metabolic acidosis of unknown cause, respectively. Methanol toxicity outbreak was not suspected until the encounter of the third victim, whom presented with seizure and severe metabolic acidosis. The attending emergency physician made an inquiry in the emergency physicians’ local network for any similar presentations in other nearby hospitals or known cases of methanol poisoning. We were informed that another patient had similar manifestations of severe high anion gap metabolic acidosis after recently consuming hard liquor together with a group of friends. Subsequently, we experienced a sudden influx of patients with similar presentations of metabolic acidosis of varying severities. The diagnoses of methanol toxicity outbreak were made and confirmed by serum methanol levels. Emergency and ICU medical staffs were actively involved in treating and mobilizing the patients for admission.

However, due to the surge of patients, the hospital capacity to manage these cases was overwhelmed. A surge capacity analysis of surrounding hospitals, including ICU bed availabilities, dialysis capacity and patient load, was conducted immediately on day one after the diagnoses of the third presenting victim. Subsequently, a decision was made to transfer appropriate cases to other nearby hospitals.

We performed a retrospective review of the electronic medical records of all methanol toxicity patients. A data set was established with all parameters extracted from the patients’ records. The data were compiled and analysed independently.

### Treatment management

Once the diagnosis was established, we formulated a standard management for all of the patients. Our medical and ICU teams were actively involved in managing the cases. All patients had blood investigations consisting of blood gases, capillary blood glucose, full blood count, renal profile, liver function test, amylase, serum osmolarity, lactate and electrocardiogram (ECG). Patients with severe acidosis, pH less than 7, and reduce conscious level were intubated. Patients with serum potassium of above 5 on blood gas analysis received intravenous calcium gluconate, intravenous dextrose 50% and intravenous insulin. One hundred millilitres of intravenous sodium bicarbonate 8.4% was given to buffer the acidosis in the initial period. All patients received 40% oral ethanol which was prepared by the in-house pharmacist as fomepizole is not available in our centre. The loading dose was 2.3 mL/kg followed by a maintenance dose of 0.76 mL/kg per hour. This was given through nasogastric tube which was modified. The nasogastric tube was connected to a paediatric T-piece, which was then connected to 50 mL syringe via extension tubing. Other supportive measures administered were intravenous thiamine 300 mg, intravenous folinic acid 50 mg, intravenous pantoprazole 80 mg and intravenous fluid bolus 20 mL/kg in 1 h, followed by 10 mL/kg for the next 1 h, and then further fluid boluses and maintenance based on patient’s condition. The sole decisions for haemodialysis were made by the nephrologist.

The cases were immediately notified to the Crisis Preparedness and Response Centre (CPRC), a unit under the Ministry of Health Malaysia. The CPRC together with the authority investigated all of the cases and identified the source of toxic alcohol. The authorities seized 17,374 L of counterfeit liquor in 1063 raids nationwide [11].

## Results

### Patients

**Fig. 1:**
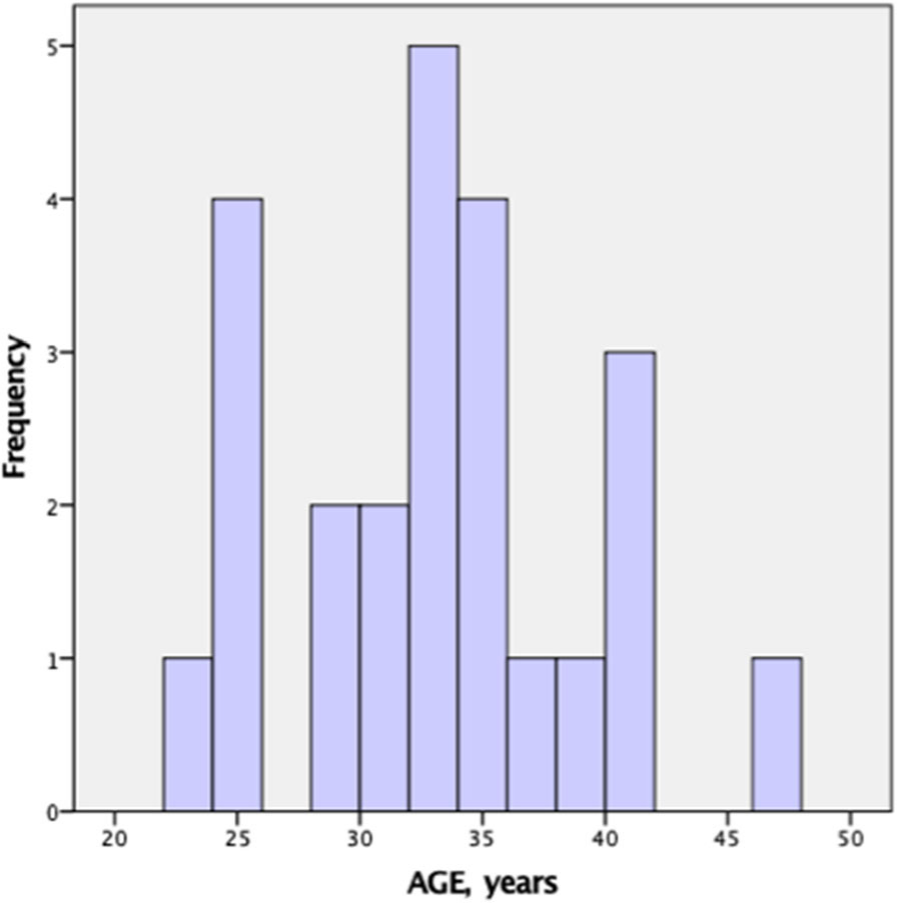
Age range of the patients

A total of 31 patients were received over the period of 9 days. Thirty of them were males with a mean age of 32 years old. Figure 1 shows the age range of the patients. They were mostly foreigners of multiple nationalities (see Table 1). From the 31 patients, 19.3% were dead on arrival, 3.2% died in the emergency department and 38.7% survived and discharged. The overall mortality rate was 61.3%. Out of the 12 patients who survived, two patients had toxic optic neuropathy, and one patient had uveitis. The rest of the survivors did not have any long-term complications.

**Table 1:** Demographic of nationality

Nationalities	Number of patients (%)
Malaysia	2 (6.5)
Bangladesh	5 (16.1)
Myanmar	3 (9.7)
India	2 (6.5)
Nepal	19 (61.3)

Looking at their clinical presentations, the last alcohol intake was within 24 to 96 h prior to presentation. From the 25 patients who came alive, 35.5% presented with vomiting, 32.3% had shortness of breath, 32.3% had blurring of vision, 29% had altered conscious level, 19.4% had abdominal pain and 6.5% had seizure. Thirteen patients were both intubated and dialysed. Out of this, 3 survived. Seven patients were intubated but not dialysed, and 4 survived. One patient was not intubated but dialysed, and he survived. The rest of the patients did not undergo invasive management and they all survived.

### Blood analysis

We also analysed the laboratory investigations of the 24 patients who were treated in our hospital. We did not include the patient who died in the department, as the initial laboratory investigations were not available given that he succumbed early.

**Fig. 2:**
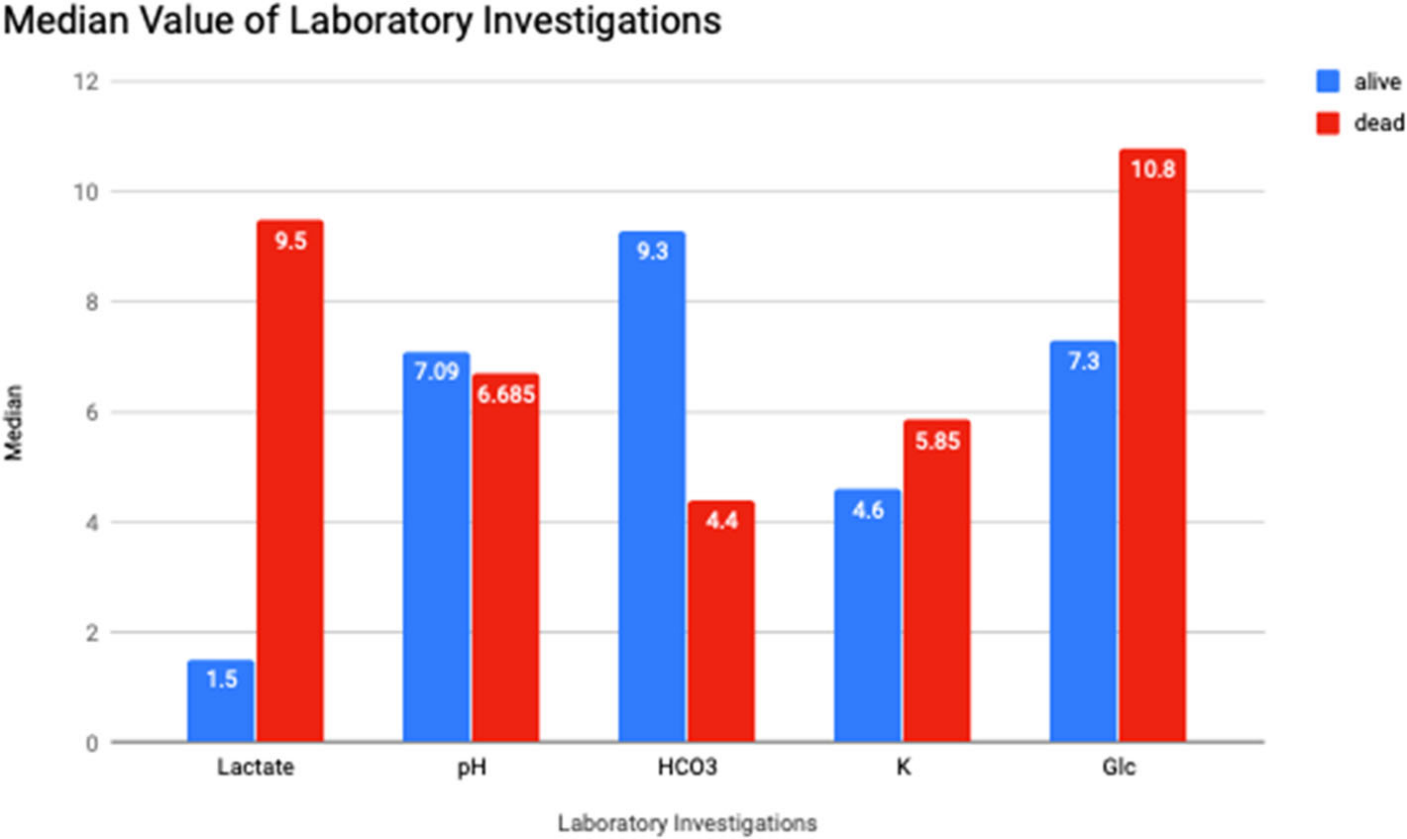
Median values of laboratory investigations

Figure 2 shows the median results of laboratory investigations comprising of blood pH, serum lactate, serum bicarbonate, serum potassium and serum glucose levels between those who survived and succumbed. Higher values of serum lactate and serum potassium were seen in patients who did not survive. The highest serum potassium recorded was 8.1 mmol/L.

**Fig. 3:**
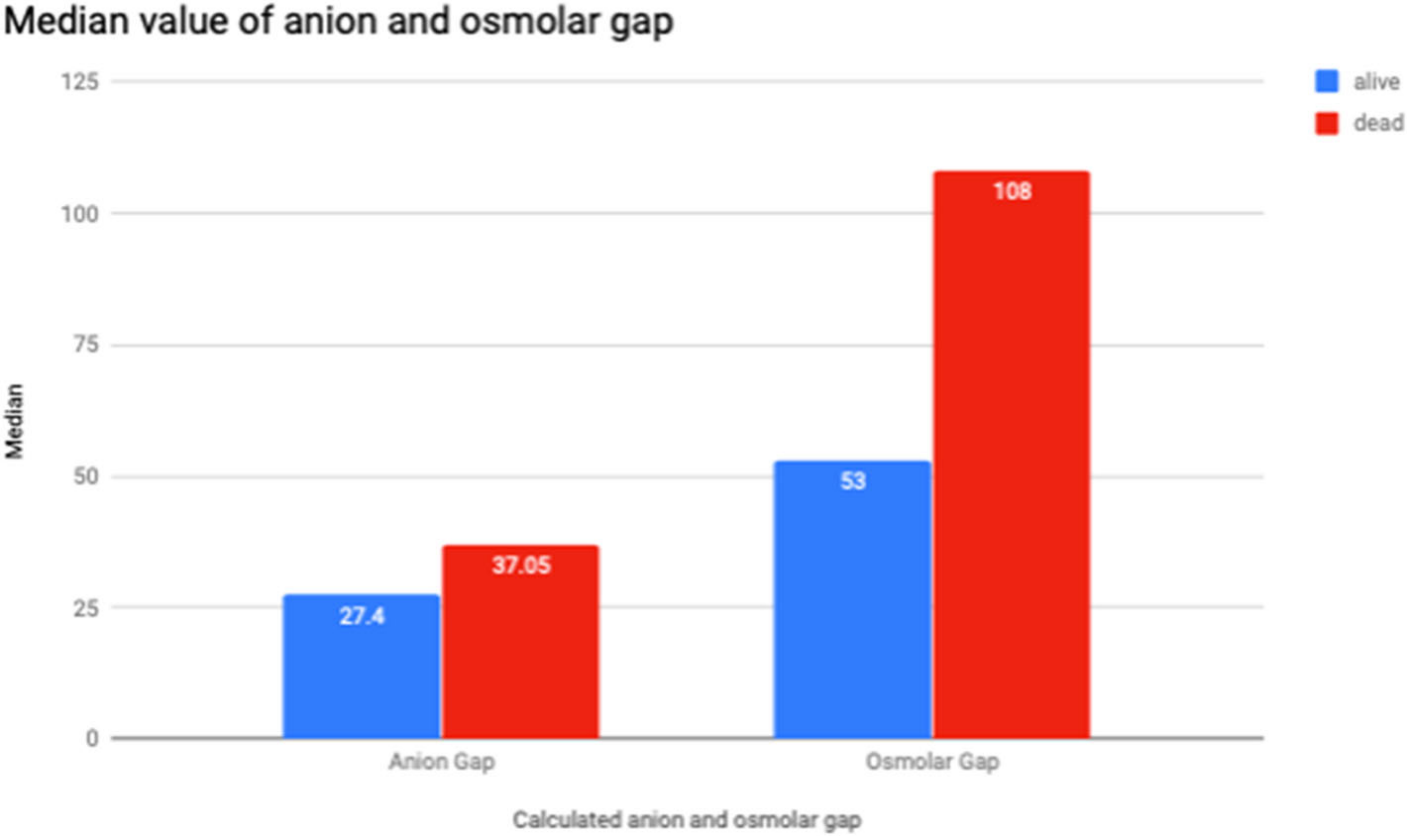
Median value of anion and osmolar gap

Figure 3 shows the differences of the calculated anion gap and osmolar gap between patients who survived and succumbed. Osmolar gap is higher in patients who died compared with patients who survived (med 108 vs 53 mOsm/L).

As our data were not normally distributed with continuous variable, we performed Mann-Whitney *U* test to look at the median between the two groups—those that survived and those that succumbed (Table 2). There were significant differences of median between serum pH, bicarbonate, lactate, potassium, anion gap, osmolar gap and measured serum osmolarity. Overall, patients that succumbed had lower pH (lowest value 6.5), lower serum bicarbonate (lowest value 0), higher serum lactate (highest value 12.8 mmol/L) and potassium (highest value 8.1 mmol/L) and higher osmolar gap (highest value 213 mOsm/L) and anion gap (highest value 73.3).

**Table 2:** Mann-Whitney *U* test comparing median between the group that survived and the group that succumbed

Laboratory blood parameters	Median	IQR	*p* value
Serum pH			
Alive	7.090	0.09	< 0.001
Dead	6.685	0.14	
Serum bicarbonate			
Alive	9.300	2.20	< 0.001
Dead	4.400	2.50	
Serum lactate			
Alive	1.500	1.60	< 0.001
Dead	9.500	4.90	
Serum potassium			
Alive	4.600	1.10	0.003
Dead	5.850	2.70	
Serum glucose			
Alive	7.300	2.10	< 0.060
Dead	10.800	8.00	
Haematocrit			
Alive	54.900	4.100	0.630
Dead	52.050	5.500	
Anion gap			
Alive	27.400	9.200	0.004
Dead	37.050	7.000	
Osmolar gap			
Alive	53.000	57.00	0.001
Dead	108.000	32.30	
Measured serum osmolarity			
Alive	330.000	55.00	0.001
Dead	397.000	47.00	
Time to haemodialysis			
Alive	13.500	57.50	0.106
Dead	9.5500	17.50	

The analysis of serum methanol level was only performed to 24 of 31 patients. The sampling was not performed on arrival. Some were performed during post-mortem. The results were only available 1 week later. Seven cases were undetected, whilst the detected levels ranged from 8 to 413 mg/dL. Due to the delay of the serum methanol results, our patients were not managed based on its level. Hence, we did not analyse this parameter and its correlation.

There is no significant difference in median for time to haemodialysis. It stands to reason that patients who were both intubated and dialysed are the most ill and therefore has poorer outcome. However, the 3 patients (patients 2, 13 and 17—see Table 3), who were intubated and dialysed and survived, had higher bicarbonate and lower lactate. Laboratory investigations, invasive management, and mortality outcomes of patients are presented in Table 3.

**Table 3:** Laboratory investigations, invasive management and mortality outcomes of patients with suspected methanol poisoning

Patient	Laboratory investigations											Invasive management		Outcome (D/A)
	pH	HCO3 (mmol/L)	Base excess (mmol/L)	Lactate (mmol/L)	Anion gap	Serum osmolarity (mOsm/L)	Osmolar gap	Serum methanol (mg/dL)	Glucose (mmol/L)	HCT (%)	K (mmol/L)	Intubation (Y/N)	Dialysis (Y/N)	
1	6.83	0	− 28.3	7.5	39.2	277	Unable to calculate	Not detected	7.2	65	5.2	Y	Y	D
2	7.05	6.1	− 22.3	4.9	36.1	286	Unable to calculate	Not detected	11	60.3	5.2	Y	Y	A
3	6.67	0	− 25.5	7.1	37.8	280	Unable to calculate	Not tested	16.1	54.7	5.8	Y	N	D
4	6.7	4	− 25	12	42.1	277	71	Not detected	7.8	56	5.1	Y	Y	D
5	6.67	6.9	− 26	9	35	291	107	Not detected	10.8	61.7	5.9	Y	Y	D
6	6.64	4.5	− 37	12.7	36.9	277	108	8	7.2	55.7	6.4	Y	Y	D
8	7	4.8	− 21	7.4	30.8	281	40	30	11	61.7	6	Y	N	A
9	6.68	4.3	− 35	8.9	37.2	282	155	45	7.7	50.1	7.5	Y	Y	D
10	7.04	8.1	− 23	2.2	22.7	269	37	Not detected	5.9	54.9	4.8	Y	N	A
13	7.09	8.8	− 21.3	3.4	35	273	93	Not tested	8.1	56.9	6.6	Y	Y	A
14	6.91	6.1	− 26.9	5.1	28.5	108	73	14	11.2	55.6	4.6	Y	Y	D
15	7.06	8.3	− 22.9	1.5	25.4	284	114	Not tested	8.8	55.6	3.7	Y	N	A
16	6.5	2.8	− 30	14	40.5	306	116	70	17.7	51.3	4.3	Y	N	D
17	7.08	10.1	− 17	1.4	31.5	300	− 15	108	7.9	5.3	3.6	Y	Y	A
18	7.22	11.7	− 13.2	5.7	29.3	294	133	149	3.3	52.8	8	Y	Y	D
19	7.18	11.4	− 13.6	1.5	20.1	292	10	Not detected	6.7	54.7	4.5	N	N	A
20	7.15	10.7	− 18.4	2.4	26.7	263	95	Not detected	7.3	51.3	5.4	N	N	A
22	6.69	4.2	− 27	10	36.6	292	104	124	20.4	45.3	5.8	Y	Y	D
23	7.18	11	− 19.6	1.9	29.8	291	1	162	6	51.1	3.8	N	N	A
24	7.11	9.3	− 20.8	1.2	73.3	284	37	78	6	56.7	4.6	N	Y	A
25	7.14	9.9	− 20.3	1.1	27.4	277	53	106	7.2	52.3	4.3	N	N	A
26	6.54	3.2	− 29	12.8	40.9	293	213	191	14.8	50.1	8.1	Y	Y	D
27	7.34	23	0.8	4.5	16.4	290	Unable to calculate	22	5.4	39.3	3.4	N	N	A
31	6.83	0	− 28.3	7.5	39.2	277	Unable to calculate	Not detected	7.2	65	5.2	Y	Y	D

*Y* yes, *N* no, *A* alive, *D* died

## Discussion

Methanol toxicity continues to interest clinical toxicologist and emergency physicians alike as initial diagnosis can be challenging, and mortality remains high despite aggressive treatment. In our experience, the majority of the patients were foreigners. This posed difficulty in getting history due to language barrier. Apart from that, the clinical presentations were not forthright. These were the cause of delay in establishing methanol outbreak. Having said that, the CPRC and the authority were quick to curb this problem, and this helped to reduce the number of patients and length of outbreak, which was 13 days.

Our hospital had limited resources to manage methanol poisoning and adhere to the recommended management. Serum methanol, serum ethanol and serum formic acid were not immediately available. These tests were only conducted at our National Laboratory Centre, and the results were available after 24 to 48 h. Fomepizole is costly and therefore not used. With that, all of our patients were clinically diagnosed by their symptoms, such as abdominal pain, vomiting, reduced consciousness and blurring of vision, or blindness with history of recent cheap liquor consumption.

The management was largely improvised according to our available resources. Ethanol level should ideally be monitored every 1 to 2 h in the initial period to ensure the serum concentration remains in the recommended therapeutic range of between 100 and 150 mg/dL. This is to prevent metabolism of methanol to formic acid which occurs when the serum ethanol concentration falls below 100 mg/dL [10]. However, as intravenous ethanol was not available, we resorted to oral ethanol. The usage of 40% oral ethanol and its dosage were advised by the pharmacist. As serum ethanol level was not available, we used base excess and lactate as biochemical surrogates and aimed to dialyse the patients as soon as possible.

All our patients were diagnosed clinically through a combination of symptoms, together with severe metabolic acidosis, high osmolar gap or high anion gap. This is consistent with the previous study which showed a linear correlation between the osmolar gap and serum methanol, and anion gap with serum formic acid. The study also suggested an osmolar gap > 25 mosmol/kg H_2_O has high specificity for early phase of methanol poisoning [12]. All our patients had high osmolar gap. But we also noted, it was markedly higher in the group that succumbed, median of 108 mosmol/kg H_2_O. Whereas the patients that survived had median osmolar gap of 53 mosmol/kg H_2_O.

Several studies have described methanol outbreak in their region. However, only few have described the correlation between outcome and laboratory parameters upon admission, which were low pH, serum ethanol and creatinine level [13–15]. Our study has found an extension to this. In patients that succumbed, besides having lower serum pH and serum bicarbonate, the serum lactate, potassium and osmolar gap were significantly higher. The high lactate can possibly be explained as follows. Firstly, the acidosis caused by accumulation of formic acid induces circulatory failure leading to tissue hypoxia and lactic acid production [16]. Secondly, formic acid inhibits the activity of cytochrome oxidase in the mitochondria, inhibiting the oxidative metabolism and hindering mitochondrial respiration. This also leads to acidosis and lactate accumulation [17].

There was a diagnostic dilemma in one patient and he was not dialysed early. However, the serum methanol later came back as high, and at this point, the decision for dialysis was made and the patient survived. This highlights that the time to haemodialysis has no correlation with patient outcome. In the analysis of our patients, haemodialysis only has weak correlation with the outcome, whilst time to dialysis does not have any correlation at all. These findings are in contrast to the report by Kute et al. [18]

The mortality for methanol poisoning ranged between 28 and 48% [4, 14, 15]. Our mortality is outside this range at 67.7%. Six out of 21 patients that died was dead on arrival or arrested in our emergency department. Four patients died after more than 2 weeks of hospitalization. These patients were complicated by hospital-acquired infection, although their acidosis improved. Eleven patients succumbed after 1 week of admission due to primary cause. We feel that the limited resources, such as unavailability of intravenous ethanol and/or fomepizole, possibly contribute to this high mortality rate, along with difficulty making initial diagnosis and late presentations.

## Limitations

We assumed the diagnosis of methanol toxicity based on history of alcohol consumptions, clinical presentations accompanied by metabolic acidosis. The data suffer from lack of certain laboratory investigation and confirmation of serum methanol level for some patients. Metabolic acidosis can also be caused by other toxicants.

## Conclusion

This paper describes a methanol poisoning outbreak, our difficulties in managing a large number of patients with limited resources and the laboratory parameters between the patients that survived and those that did not. Our mortality rate was higher compared with others and the morbidity includes permanent visual impairment and severe neurological sequelae. Language barrier, severity of illness, late presentation, unavailability of intravenous ethanol and fomipezole and delayed dialysis may have been the contributing factors. This study not only found significant differences in median for pH, serum bicarbonate, osomolar and anion gap, but also found significant differences in median for serum lactate and potassium. This was not described in previous report. We suggest for a well-designed guideline which adapts to the local resources along with collaborations with local authority to curb these large-scale methanol outbreaks in the future.

## Data Availability

Data and materials are with authors and are available upon request.

## Abbreviations

CPRC: Crisis Preparedness and Response Centre
ECG: Electrocardiogram
ED: Emergency department
ICU: Intensive care unit
